# BMP signaling is a therapeutic target in ovarian cancer

**DOI:** 10.1038/s41420-020-00377-w

**Published:** 2020-12-05

**Authors:** Tomohiko Fukuda, Risa Fukuda, Ryo Tanabe, Daizo Koinuma, Hiroo Koyama, Yoshinobu Hashizume, Aristidis Moustakas, Kohei Miyazono, Carl-Henrik Heldin

**Affiliations:** 1 Department of Medical Biochemistry and Microbiology, Science for Life Laboratory, Box 582, Uppsala University, SE-751 23 Uppsala, Sweden; 2 Department of Molecular Pathology, Graduate School of Medicine, The University of Tokyo, Hongo 7-3-1 Bunkyo-ku, Tokyo, 113-8655 Japan; 3 Drug Discovery Chemistry Platform Unit, RIKEN Center for Sustainable Resource Science, 2–1 Hirosawa, Wako, Saitama, 351–0198 Japan; 4 RIKEN Program for Drug Discovery and Medical Technology Platforms, 2–1 Hirosawa, Wako, Saitama, 351–0198 Japan

**Keywords:** Gynaecological cancer, Gynaecological cancer

## Abstract

BMP signaling has been found to have tumor-promoting as well as tumor-suppressing effects in different types of tumors. In this study, we investigated the effects of BMP signaling and of BMP inhibitors on ovarian cancer (OC) cells in vitro and in vivo. High expression of BMP receptor 2 (BMPR2) correlated with poor overall survival of OC patients in the TCGA dataset. Both BMP2 and BMPR2 enhanced OC cell proliferation, whereas BMP receptor kinase inhibitors inhibited OC cell growth in cell culture as well as in a mouse model. BMP2 also augmented sphere formation, migration, and invasion of OC cells, and induced EMT. High BMP2 expression was observed after chemotherapy of OC patients in the GSE109934 dataset. In accordance, carboplatin, used for the treatment of OC patients, increased BMP2 secretion from OC cells, and induced EMT partially via activation of BMP signaling. Our data suggest that BMP signaling has tumor-promoting effects in OC, and that BMP inhibitors might be useful therapeutic agents for OC patients. Considering that carboplatin treatment augmented BMP2 secretion, the possibility to use a combination of BMP inhibitors and carboplatin in the treatment of OC patients, would be worth exploring.

## Introduction

Ovarian cancer (OC) is the most lethal gynecologic cancer with an increasing incidence; 295,414 new cases and 184,799 deaths worldwide were reported in 2018^1^. Epithelial OC is the most common type, 70% of which consists of high-grade serous carcinoma^2^. Because OC easily disseminates intraperitoneally, many OC patients are diagnosed at advanced stages. Combination of surgery and chemotherapy is a standard treatment. However, OC often recurs even if patients show complete response to initial treatment. Although cancer stem cells and epithelial-mesenchymal transition (EMT) are likely to be involved in the recurrence, the detailed mechanisms remain to be elucidated^3–7^.

Bone morphogenetic proteins (BMPs) belong to the transforming growth factor-β (TGF-β) family. BMPs perform pivotal roles in the morphogenesis of different organs, including musculoskeletal, cardiovascular and neural systems^8^. BMP ligands bind to two types of cell surface kinase-associated receptors called type I and type II receptors. The type I BMP receptors include ALK1, ALK2, ALK3 and ALK6 (also called ACVRL1, ACVR1, BMPR1A and BMPR1B, respectively), whereas the type II BMP receptors include ACVR2A, ACVR2B, and BMPR2. Upon ligand-binding, type II receptors phosphorylate and activate type I receptors to induce phosphorylation of SMAD1/5/8. Phosphorylated SMAD1/5/8 translocates to the nucleus and activate transcription after forming complexes with SMAD4^8^. The ID family transcription factors are well-known downstream targets of BMP signaling^9–12^. In glioblastoma, BMP is a tumor suppressor by inducing differentiation^13–15^. On the other hand, BMP promotes the development of colon cancer, partially via inhibition of apoptosis^16,17^. In OC, BMP signaling has been reported to play tumor-promoting roles by enhancing cell proliferation, and has been found to be associated with poor prognosis^18,19^. BMP4 has been reported to induce EMT and stemness in OC cells^20,21^, and autocrine BMP9 promotes OC cell growth^22^. Thus, BMP signaling is an interesting pathway that can potentially be targeted in OC.

At present, a combination of platinum-based carboplatin (CBDCA) and paclitaxel is the first-line chemotherapy for OC. Once OC patients become resistant to platinum-based drugs, only limited treatment options remain. Since the promotion of cancer stem cells and EMT are related to resistance, novel therapeutic drugs limiting the numbers of cancer stem cells and the potency of EMT may be useful.

The goal of this study was to examine whether BMP signaling induces stemness and EMT in OC cells and to evaluate the effects of BMP inhibitors on OC in vitro and in vivo. In addition, these experiments led to the discovery that CBDCA can activate BMP signaling in OC cells.

## Results

### The BMP signaling pathway is activated in OC

**Fig. 1:**
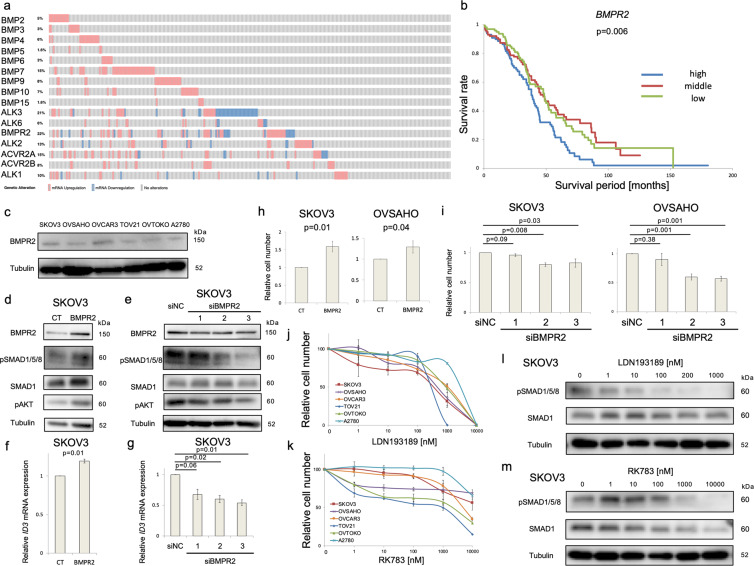
The BMP pathway is activated in ovarian cancer. **a** Expression of mRNAs for BMP ligands and receptors in 306 OC patients. RNA-seq of TCGA serous ovarian cancer dataset was analyzed via cBioPortal. RNA expression cutoff Z score was adjusted to 2.0. **b** Correlation between *BMPR2* mRNA expression and overall survival of 306 OC patients derived from the TCGA serous ovarian cancer dataset. Based on *BMPR2* mRNA expression, the 306 patients were equally divided into three groups (high, middle, low). A *P*-value was calculated with a log-rank test. **c** BMPR2 protein expression was evaluated by immunoblotting (IB) in 6 OC cell lines. **d** SKOV3 cells were transfected with an empty (CT) or BMPR2-encoding plasmid for 72 h. The expression of indicated proteins was assessed by IB. **e** SKOV3 cells were treated with a Negative Control siRNA (siNC) and three different siBMPR2 for 72 h. The expression of indicated proteins was evaluated by IB. **f** *ID3* mRNA expression of SKOV3 cells transfected with CT and BMPR2 plasmid for 72 h was analyzed by RT-PCR. **g** *ID3* mRNA expression of SKOV3 cells treated with siNC and three different siBMPR2 for 72 h was assessed by RT-PCR. **h** SKOV3 and OVSAHO cells were transfected with CT or BMPR2 plasmid for 48 h, and thereafter cells were plated in 96-well plates and incubated for an additional 48 h. Cell viability was determined by MTS assay after adjusting CT to 1. **i** After 48 h treatment with siNC or three different BMPR2 siRNAs, SKOV3 and OVSAHO cells were cultured in 96-well plates for 48 h. MTS assay was used to assess cell viability relative to siNC. **j**, **k** Six ovarian cancer cells were treated with DMSO or different concentrations of LDN193189 (**j**) or RK783 (**k**) for 72 h. MTS assay was used to analyze cell numbers relative to DMSO treatment. **l**, m SKOV3 cells were incubated with DMSO or different concentrations of LDN193189 (LDN) (**l**) or RK783 (RK) (**m**) for 24 h. IB was used to analyze the expression of indicated proteins. The results in (**f**–**i**) are shown as the mean ± SE.

The expression of mRNA for most BMP ligands and receptors was found to be frequently increased in OC by analysis of the TCGA OC database^23^ (Fig. 1a). High expression of *BMPR2* (Fig. 1b) and *BMP7* (Fig. S1a) mRNA significantly correlated with poor overall survival, whereas the other BMP ligands and receptors analyzed in this dataset did not show significant correlations (Fig. S1a, b). We validated these observations using six OC cell lines. BMPR2 protein (Fig. 1c) and mRNA (Fig. S2a) were detected in all six cell lines. *ALK3* mRNA was most abundantly expressed among the type I receptors, whereas *BMPR2* mRNA was most abundant among the type II receptors (Fig. S2a). To elucidate the function of BMPR2 in OC, it was overexpressed by transfection or silenced by siRNA in SKOV3 cells (Figs. 1d–g, S2b–e). Phosphorylation of SMAD1/5/8 and AKT, two downstream mediators of BMP signaling, and expression of the downstream gene *ID3* were induced by BMPR2 overexpression and suppressed by BMPR2 knockdown (Fig. 1d–g). BMPR2 overexpression enhanced cell growth in SKOV3 and OVSAHO cells, as determined by MTS assay (Fig. 1h), whereas two out of three siRNAs targeting BMPR2 inhibited cell proliferation in both cell lines (Fig. 1i). Similar results were obtained also in other OC cell lines (Fig. S2c, d). To investigate the growth-promoting effect of BMP signaling further, OC cell lines were treated with the BMP receptor kinase inhibitors LDN193189 and RK783^24^. Both inhibitors suppressed OC cell growth in a dose-dependent manner (Fig. 1j, k), which was accompanied by suppression of SMAD1/5/8 phosphorylation (Fig. 1l, m). Complete inhibition of SMAD1/5/8 phosphorylation was obtained at 200 nM LDN193189 and 1 μM RK783; these concentrations of the inhibitors were therefore used in further experiments.

### BMP2 enhances OC cell proliferation and sphere formation via c-KIT induction

**Fig. 2:**
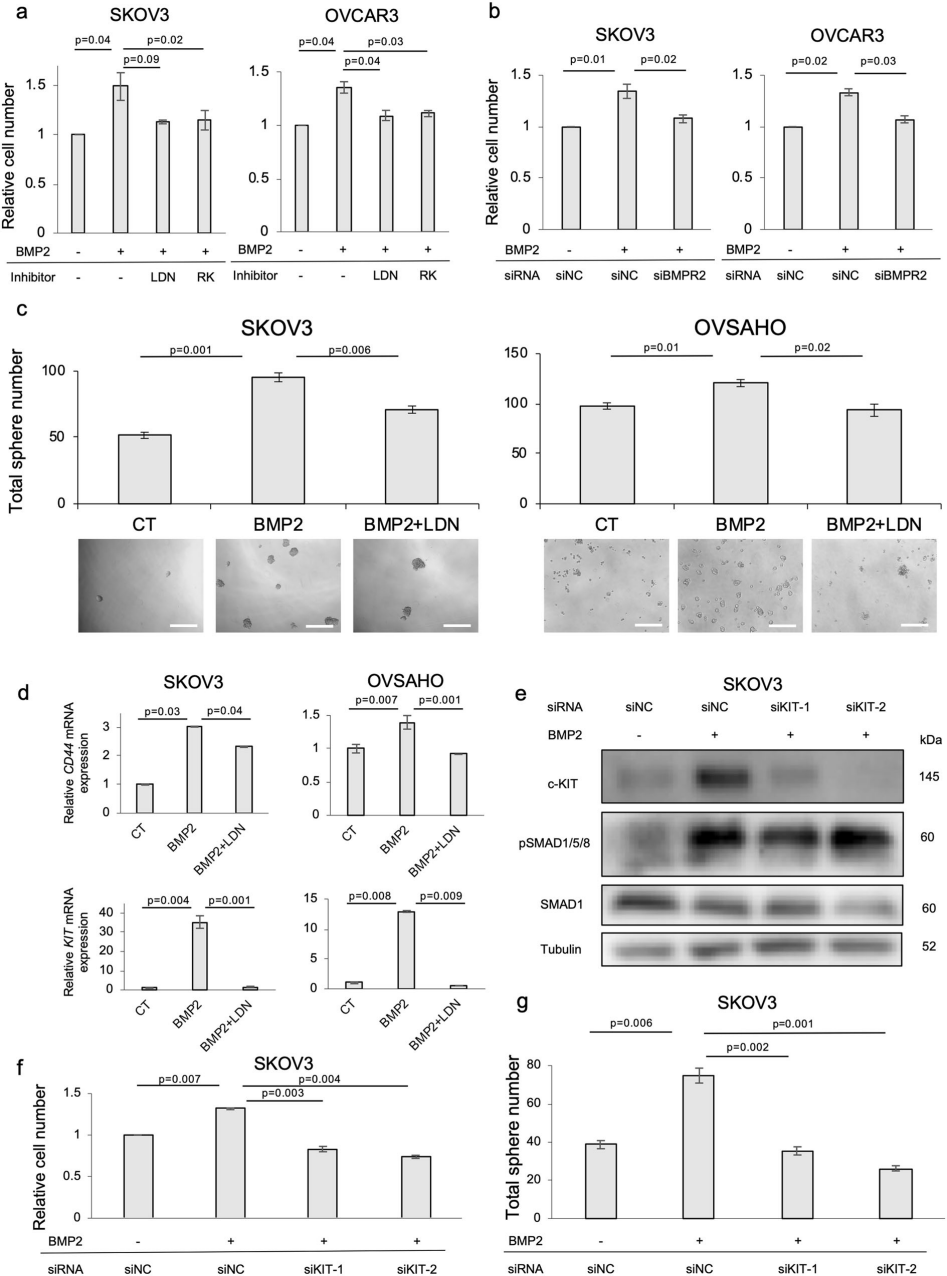
BMP2 enhances OC cell proliferation and sphere formation via c-KIT induction. **a** MTS assay was performed to monitor cell viability. SKOV3 and OVCAR3 cells were treated with PBS (CT) or 20 ng/ml BMP2 in combination with LDN193189 (LDN) or RK783 (RK) for 72 h. Cell numbers were normalized relative to CT. **b** After 48 h transfection with siNC or siBMPR2, SKOV3 and OVCAR3 cells were incubated in 96-well plates with and without 20 ng/ml BMP2 for an additional 48 h. Cell viability was assessed by MTS assay relative to siNC without BMP2. **c** Sphere formation assay was used to evaluate cancer stemness. SKOV3 and OVSAHO cells were cultured with stem cell medium containing 20 ng/ml BMP2 and LDN in 96-well ultra-low attachment plates for 8 days. Sphere numbers per well were counted under microscopy. Images of spheres are shown at bottom of each group. Scale bar = 200 µm. **d** Expression of *CD44* and *c-KIT* mRNA was analyzed by RT-PCR in SKOV3 and OVSAHO cells. Cells were treated with PBS (CT), BMP2 (20 ng/ml) or BMP2 + LDN for 72 h. mRNA expression is shown as fold change relative to CT. **e** BMP2-induced c-KIT protein expression was assessed by IB in SKOV3 cells. SKOV3 cell was pretreated with siNC or two different c-KIT siRNAs (siKIT-1, siKIT-2) for 48 h and stimulated with PBS or 20 ng/ml BMP2 for an additional 48 h. Then protein was extracted and subjected to IB. **f**, **g** After 48 h transfection with siNC or siKIT-1 or siKIT-2, collected SKOV3 cells were cultured in the presence and absence of 20 ng/ml BMP2 for an additional 48 h or 8 days for MTS assay or sphere formation assay, respectively. Cell viability was determined by MTS assay relative to siNC without BMP2. The results in (**a**–**d**) and (**f**–**g**) are shown as the mean ± SE.

The effects of stimulation of SKOV3 and OVSAHO cells with BMP2, BMP4, and BMP7 were investigated. Most pronounced SMAD1/5/8 phosphorylation was observed after BMP2 stimulation in both cell lines (Fig. S3a). In accordance with this result, BMP2-induced *ID1* and *ID3* more prominently than BMP4 in SKOV3 cells (Fig. S3b). As expected, BMP2 promoted proliferation of SKOV3 and OVCAR3 cells and the BMP receptor kinase inhibitors, LDN193189 and RK783, inhibited the effect (Fig. 2a). Moreover, BMPR2 knockdown also attenuated BMP2-induced cell growth in both cell lines (Fig. 2b). In addition, BMP2 significantly enhanced sphere formation in three OC cell lines, whereas LDN193189 canceled the effect (Figs. 2c, S3c). mRNAs for the stem cell-associated transcription factors, SOX2, OCT4, and NANOG, were significantly upregulated after BMP2 stimulation in SKOV3 cells (Fig. S3d), as were the expressions of mRNA for the cell surface cancer stem cell markers CD44 and c-KIT^25–29^ in SKOV3 and OVSAHO cells; expressions of CD44 and c-KIT were suppressed in the presence of LDN193189 (Fig. 2d). The effect of c-KIT on cell growth and stemness was investigated by knocking down c-KIT by two siRNAs in SKOV3 cells. The induction of c-KIT by BMP2 and its suppression by siRNAs was confirmed by immunoblotting (Fig. 2e). c-KIT knockdown neutralized enhanced cell proliferation and sphere formation after BMP2 stimulation in SKOV3 cells (Fig. 2f, g). In addition, pharmacological inhibition of c-KIT by imatinib attenuated BMP2-induced cell growth and sphere formation in SKOV3 and OVCAR3 cells (Fig. S3f, g). These results suggest that BMP2 promotes OC proliferation and stemness via c-KIT induction.

### BMP2 induces EMT of OC cells and enhances migration and invasion

**Fig. 3:**
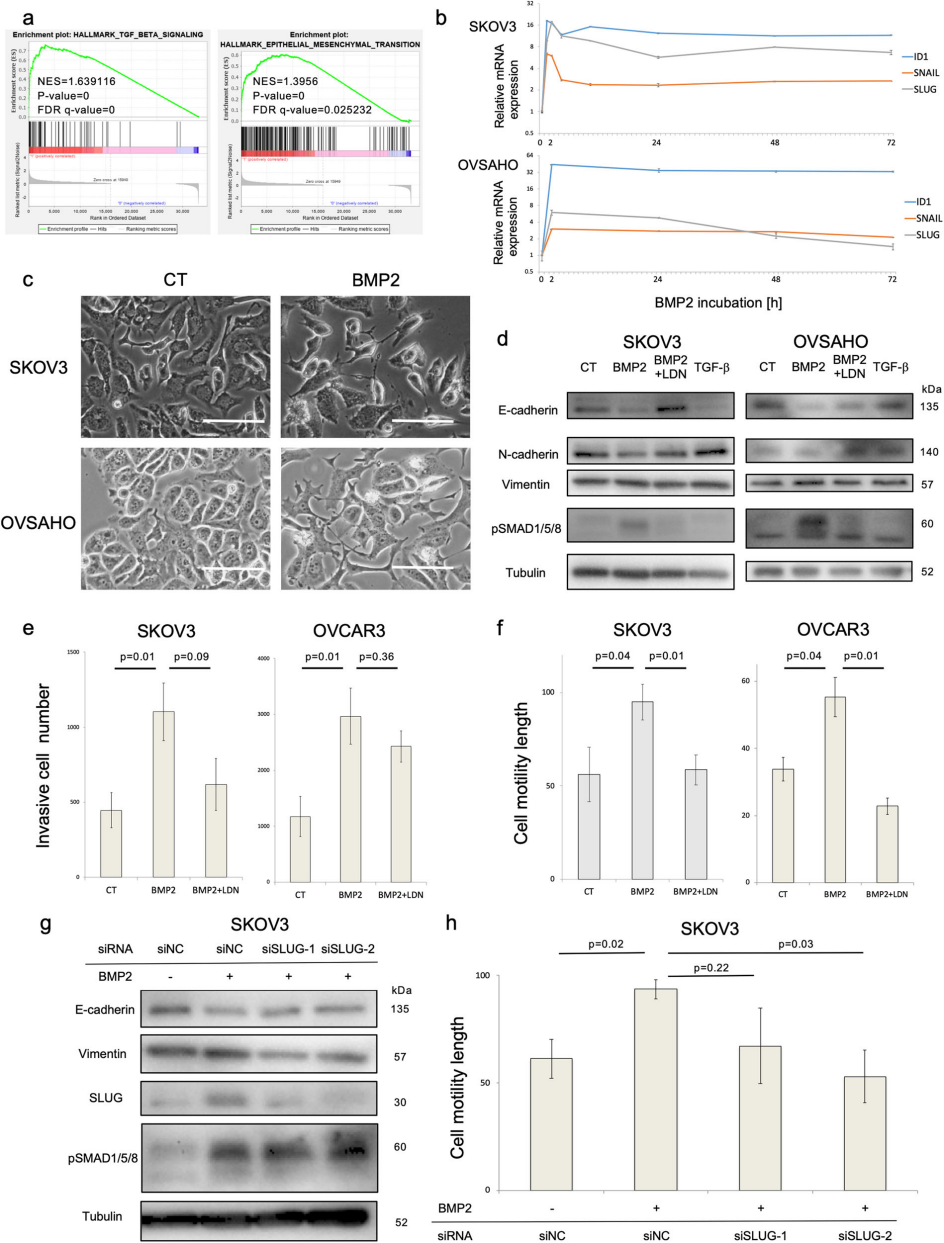
BMP2 enhances OC cell migration and invasion via EMT induction. **a** GSEA of RNA-seq was conducted in SKOV3 cells. SKOV3 cells were treated with PBS or 20 ng/ml BMP2 for 2 h and total RNAs were extracted. The left panel shows the enrichment plot of hallmark-TGF-β signaling, whereas the right panel shows that of hallmark-EMT. **b** Transition of *SNAIL*, *SLUG*, and *ID1* mRNA expression after BMP2 stimulation was analyzed by RT-PCR. SKOV3 and OVSAHO cells were cultured in serum-free medium overnight and treated with 20 ng/ml BMP2 for indicated time periods. mRNA expression was normalized relative to 0 h and shown on a logarithmic scale. **c** Micrograph of the comparison of cell morphology between SKOV3 and OVSAHO cells treated with PBS (CT) or 20 ng/ml BMP2 for 72 h. Scale bar = 25 µm. **d** SKOV3 and OVSAHO cells were incubated with PBS, 20 ng/ml BMP2, BMP2 + LDN, or 5 ng/ml TGF-β in 1% FBS-containing medium for 72 h. Indicated proteins, including EMT markers, were evaluated by IB. **e** Cell invasion was assessed by transwell invasion assay. SKOV3 and OVCAR3 cells were pretreated with PBS, 20 ng/ml BMP2 or BMP2 + LDN in 1% FBS-containing medium for 24 h and applied to upper chambers. Complete medium was added to lower chambers. Invasive cell numbers were calculated using standard curves. **f** Cell migration was evaluated by a scratch assay. Confluent SKOV3 and OVSAHO cell cultures were scratched by 100 µl pipette tips and incubated for 48 h. Cell motility length was determined by comparing the gaps between the cell sheets at 0 and 48 h. (g,h) SKOV3 cells were transfected with siNC or two different SLUG siRNAs for 48 h, followed by treatment with PBS or 20 ng/ml BMP2 in DMEM containing 1% FBS for an additional 48 h. Indicated proteins were detected by IB, whereas cell migration was assessed by a scratch assay, as previously mentioned. The results in (**e**), (**f**), and (**h**) are shown as the mean ± SE.

GSEA of RNA-sequencing was used to elucidate the effect of BMP2 in SKOV3 cells. BMP2 significantly enhanced hallmark TGF-β signaling and EMT gene sets in SKOV3 cells (Fig. 3a). Affected genes were also related to cardiac EMT and cushion formation (Fig. S4a). In agreement with this result, mRNA expression of *SLUG* and *SNAIL*, encoding EMT transcription factors, was promoted by BMP2 in SKOV3 and OVSAHO cells in a time-dependent manner, and suppressed by the type I receptor kinase inhibitor LDN193189 (Fig. 3b, S4b, S4c). Morphological changes were observed after BMP2 stimulation in both cell lines, possibly indicating induction of a partial mesenchymal-like phenotype (Fig. 3c). Immunoblotting of EMT-related proteins revealed that the epithelial marker E-cadherin was commonly suppressed by BMP2, which was reverted by LDN193189 (Fig. 3d). The mesenchymal markers N-cadherin and vimentin were already expressed and were not appreciably affected by BMP2 and the well-established EMT-inducer, TGF-β, gave very weak responses in these cells (Fig. 3d). Moreover, BMP2-stimulated OC cell invasion and migration, as determined by invasion and scratch assays, and LDN193189 neutralized these effects (Fig. 3e, f). Since SLUG induction was most evident after BMP2 stimulation (Fig. 3b), we knocked down SLUG by two different siRNAs in SKOV3 cells. SLUG knockdown reversed the suppression of E-cadherin induced by BMP2 and attenuated the expression of the mesenchymal marker vimentin (Fig. 3g), and inhibited BMP2-induced cell migration in SKOV3 cells (Fig. 3h). These results suggest that BMP2 enhances EMT, migration and invasion of OC cells in a SLUG-dependent manner.

### RK783 inhibits OC cell growth in vivo

**Fig. 4:**
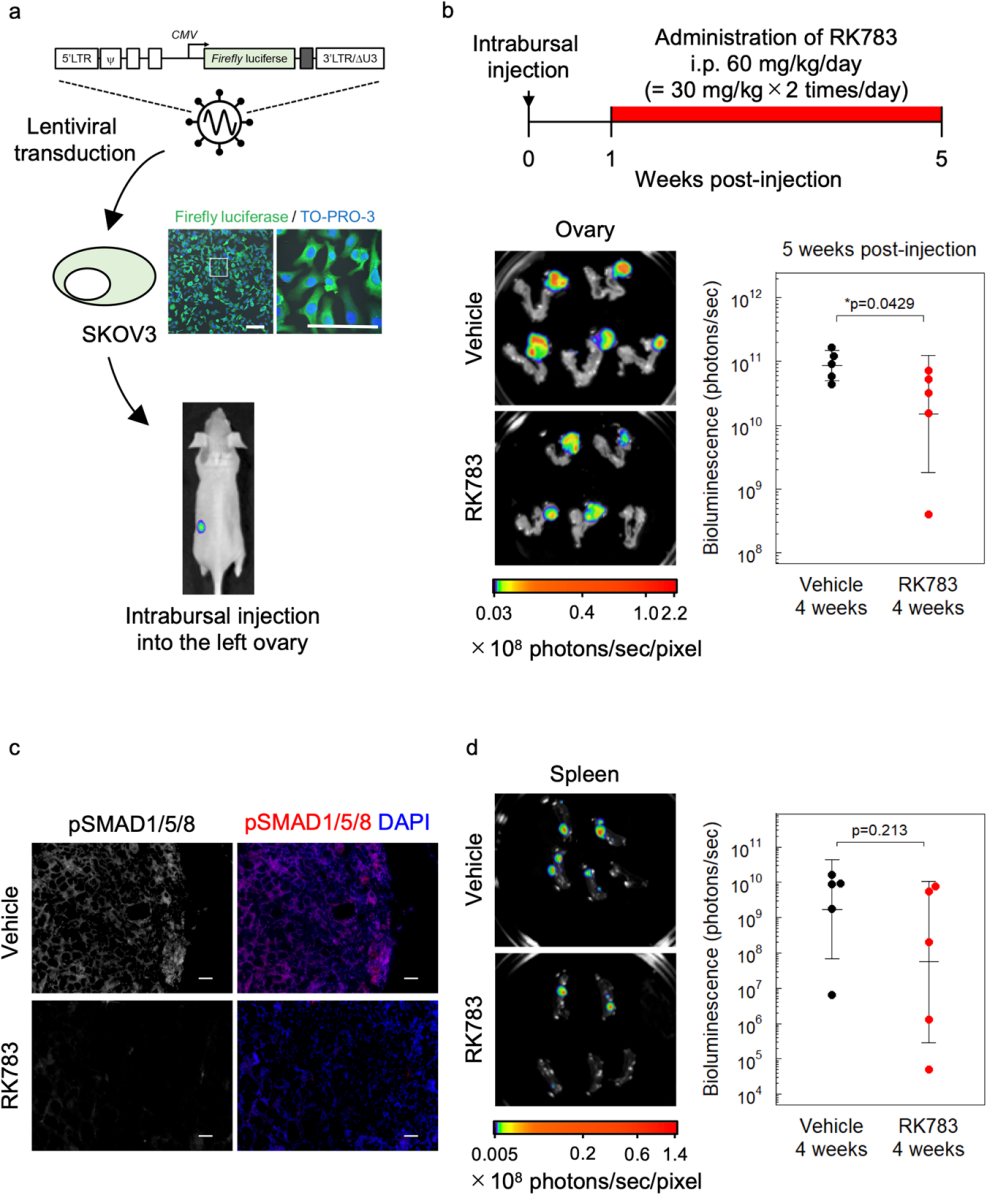
RK783 inhibits OC cell growth in vivo. **a** Generation of orthotopic xenograft of SKOV3 cells. *Firefly* luciferase gene driven by CMV promoter was lentivirally transduced into SKOV3 cells. Cells were orthotopically inoculated into the left ovary of BALB/c *nu/nu* mice. Scale bar = 25 µm. **b** Ex vivo bioluminescence imaging of orthotopic xenograft of SKOV3 cells at 4 weeks post-treatment of RK783 (*n* = 5 mice per group). **c** Phosphorylation status of SMAD1/5/8 in orthotopic xenograft of SKOV3 cells at 4 weeks post-treatment of RK783 or vehicle, as determined by immunofluorescent staining. Scale bar = 50 µm. **d** Ex vivo bioluminescence imaging of spleen derived from the orthotopic xenograft of SKOV3 cells. The results are shown as the mean ± SD.

To investigate whether BMP signaling has tumorigenic functions in vivo, we established an orthotopic xenograft mouse model using SKOV3 cells expressing the *firefly* luciferase gene (Fig. 4a). After inoculation of the SKOV3 cells into the left ovary of BALB/c *nu/nu* mice, the mice were intraperitoneally injected with vehicle or the type I receptor kinase inhibitor RK783 (30 mg/kg, twice a day) for 4 weeks. In this orthotopic model, RK783 significantly suppressed OC cell growth in vivo (Fig. 4b). Suppression of SMAD1/5/8 phosphorylation by RK783 treatment in vivo was confirmed by immunofluorescent staining of tumor sections (Fig. 4c). In addition, OC dissemination to the spleen tended to be inhibited by RK783 (Fig. 4d), although this effect was not statistically significant. Metastases to other organs were not significantly suppressed by RK783 treatment (Fig. S5). These results suggest that BMP signaling enhances ovarian tumor growth in vivo and that RK783 may be used as a chemotherapeutic drug for the treatment of OC patients.

### Chemotherapy activates BMP signaling in OC

**Fig. 5:**
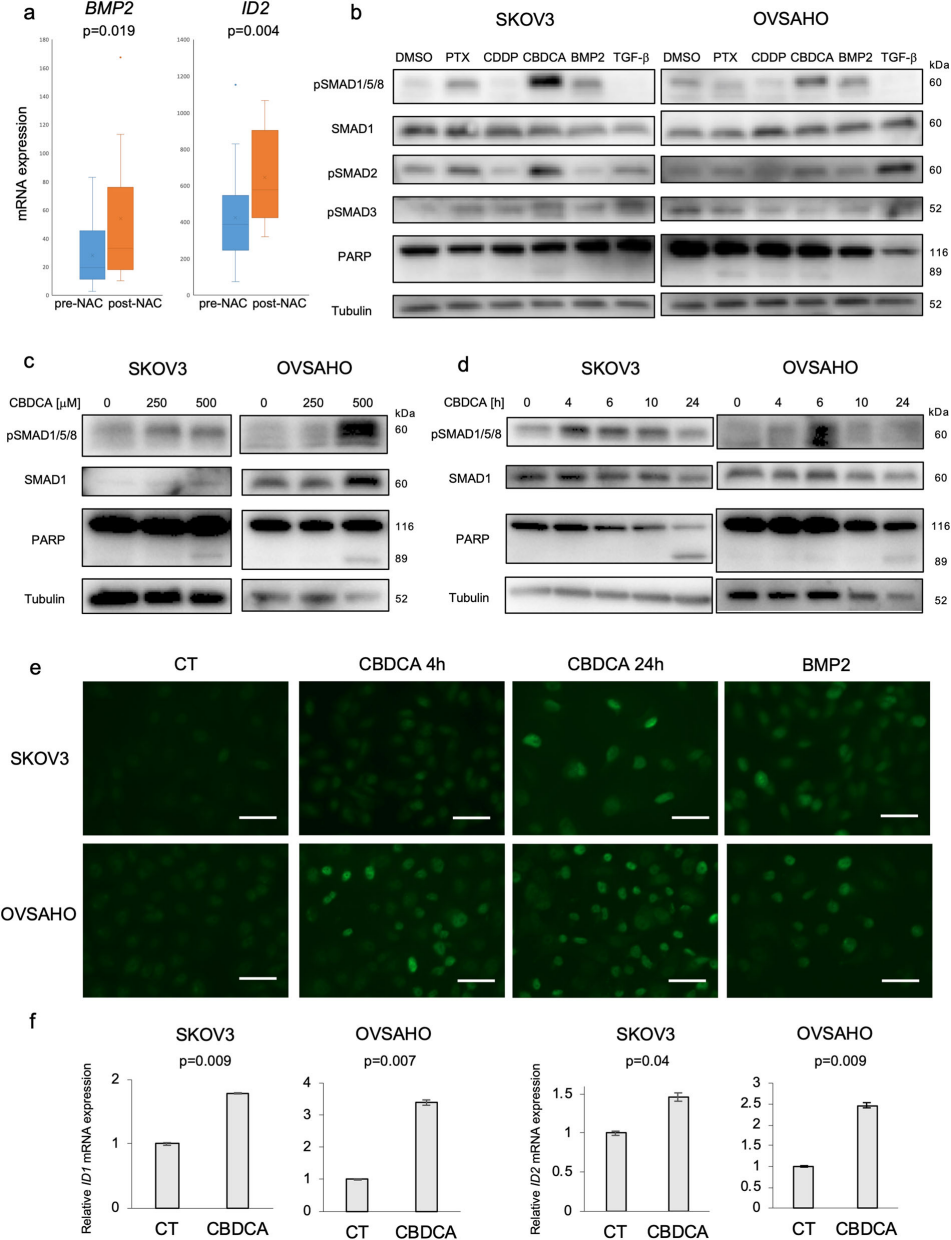
Chemotherapy activates BMP signaling in ovarian cancer. **a** The GSE109934 dataset which included RNA-seq of 19 pre-chemotherapeutic (pre-NAC) and 19 post-chemotherapeutic (post-NAC) OC patients was used to assess BMP2 and ID2 mRNA expression. Data analysis was performed using the GEO2R tools. **b** SKOV3 and OVSAHO cells were treated with DMSO, 10 nM paclitaxel (PTX), 50 µM cisplatin (CDDP), 500 μM carboplatin (CBDCA), 20 ng/ml BMP2 and/or 5 ng/ml TGF-β for 24 h. IB was performed to detect indicated proteins. **c** Dose-dependent SMAD1/5/8 phosphorylation was assessed by IB in SKOV3 and OVSAHO cells incubated with 0, 250, 500 µM CBDCA for 24 h. **d** SMAD1/5/8 phosphorylation was evaluated using IB of SKOV3 and OVSAHO cells treated with 500 µM CBDCA for indicated time periods. **e** Immunofluorescent staining was performed to detect the nuclear accumulation of phospho-SMAD1/5/8. SKOV3 and OVSAHO cells were treated with PBS or 500 µM CBDCA for 4 or 24 h, or 20 ng/ml BMP2 for 2 h. After fixation, cells were stained with a phospho-SMAD1/5/8 antibody. Scale bar = 10 µm. **f** ID1 and ID2 mRNA induction by CBDCA was analyzed by RT-PCR. SKOV3 and OVSAHO cells were treated with or without (CT) 500 µM CBDCA for 24 h. mRNA expression was normalized to CT. The results in (**f**) are shown as the mean ± SE.

We explored the possibility that chemotherapy activates BMP signaling in OC patients. Analysis of a public data set (GSE109934) proved that post-chemotherapeutic (post-NAC) patients had significantly higher *BMP2* and *ID2* mRNA expression than pre-chemotherapeutic (pre-NAC) patients (Fig. 5a). To investigate the effect of chemotherapy in vitro, SKOV3 and OVSAHO cells were treated with CBDCA, paclitaxel (PTX), and cisplatin (CDDP), which are representative chemotherapeutic drugs used in the treatment of OC patients. In both cell lines, CBDCA strongly enhanced SMAD1/5/8 phosphorylation, PTX showed weaker effects, whereas CDDP slightly attenuated SMAD1/5/8 phosphorylation (Fig. 5b). CBDCA also augmented SMAD2 phosphorylation, but only in SKOV3 cells (Fig. 5b), and as described earlier (Fig. 3d), the overall impact of TGF-β in these cells is poor. Dose- and time-dependent phospho-SMAD1/5/8 induction by CBDCA was confirmed in three OC cell lines (Figs. 5c, d, S6a, S6b). In all experiments with the chemotherapeutic drugs, the onset of cytotoxicity in vitro was also confirmed by monitoring PARP-1 cleavage (Fig. 5b–d). Immunofluorescent staining revealed that CBDCA-induced nuclear accumulation of phospho-SMAD1/5/8 in SKOV3 and OVSAHO cells, to a comparable or even higher degree compared to the BMP2-stimulated positive control (Fig. 5e). In addition, the BMP downstream genes, *ID1* and *ID2*, were significantly upregulated after CBDCA treatment of OC cells (Figs. 5f, S6c, S6d). These results suggest that chemotherapy, especially CBDCA, activates BMP signaling in OC.

### CBDCA induces EMT in OC cells, partially via BMP signaling

**Fig. 6:**
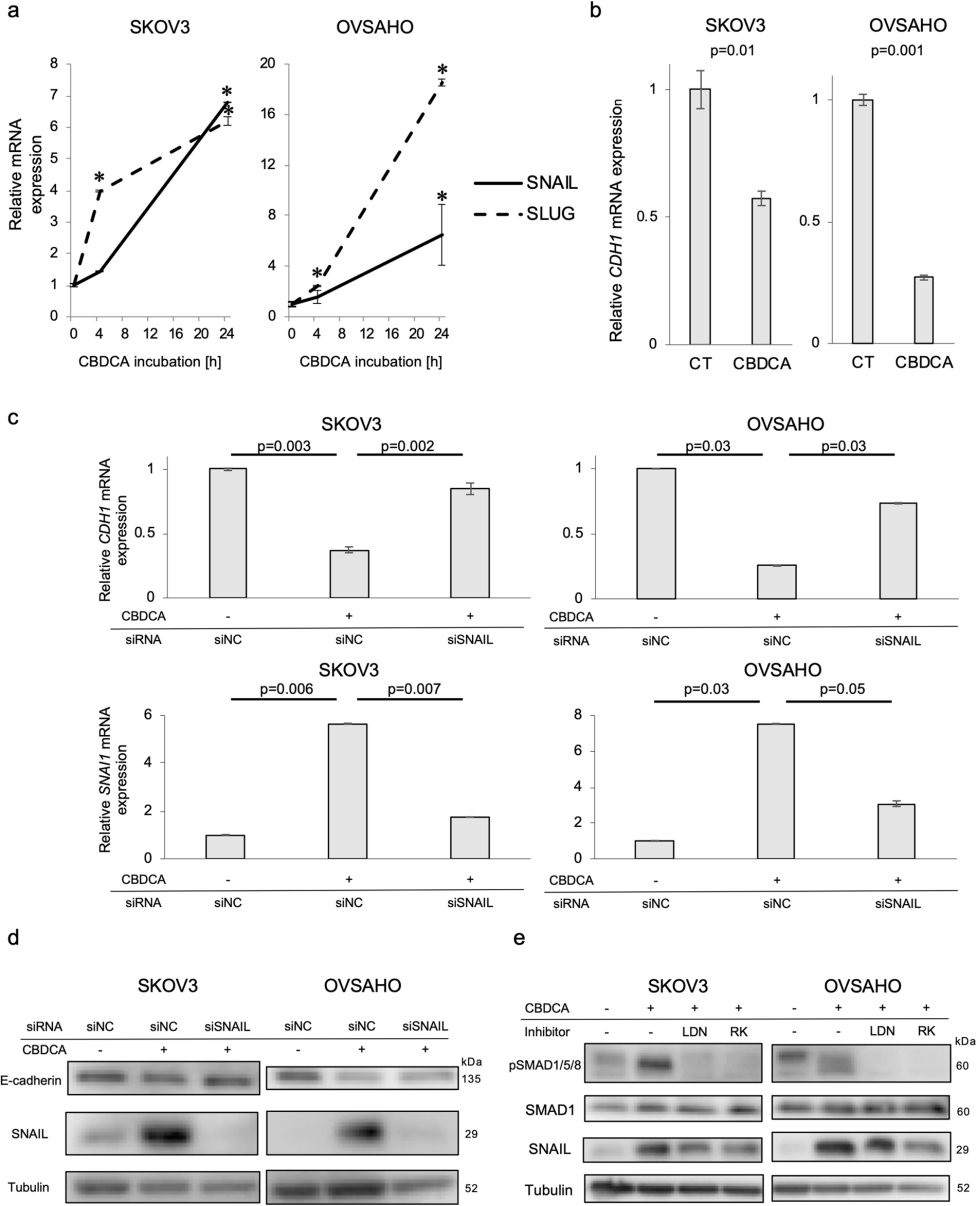
CBDCA induces EMT partially via BMP signaling in ovarian cancer cells. **a** Time-dependent *SNAIL* and *SLUG* mRNA induction by CBDCA was assessed by RT-PCR. SKOV3 and OVSAHO cells were treated with 500 µM for indicated time periods. mRNA expression was compared to no treatment group. *P-value < 0.05. **b** *CDH1* (E-cadherin) mRNA expression was evaluated by RT-PCR after 24-h incubation with and without 500 µM CBDCA. Relative mRNA expression to no treatment (CT) is shown. **c**, **d** SKOV3 and OVSAHO cells were transfected with siNC or SNAIL siRNA for 72 h and incubated with 500 µM CBDCA for 24 h (for RNA) or 48 h (for protein). E-cadherin (*CDH1*) and SNAIL (*SNAI1*) mRNA and protein expression were analyzed by RT-PCR and IB, respectively. **e** Attenuation of CBDCA-induced SNAIL and SMAD1/5/8 phosphorylation by BMP inhibitors was determined by IB. SKOV3 and OVSAHO cells were treated with DMSO, CBDCA, CBDCA + LDN193189 or CBDCA + RK783 for 24 h. The results in (**a**–**c**) are shown as the mean ± SE.

As BMP2-induced EMT in OC cells (Fig. 2), we investigated whether CBDCA could also induce EMT. CBDCA enhanced *SNAIL* and *SLUG* mRNA expression in SKOV3 and OVSAHO cells in a time-dependent manner (Fig. 6a). *SNAIL* induction was also observed in CBDCA-treated OVCAR3 cells, but these cells did not express *SLUG* mRNA (Fig. S6e). Furthermore, *E-cadherin* mRNA expression was suppressed by CBDCA in all three OC cells (Figs. 6b, S6f). Because *SNAIL* induction by CBDCA was observed in all three OC cell lines, we investigated the effect of knocking down SNAIL on E-cadherin expression in CBDCA-treated SKOV3 and OVSAHO cells. SNAIL knockdown restored CBDCA-suppressed E-cadherin expression at both mRNA and protein levels (Fig. 6c, d), indicating that CBDCA-suppressed E-cadherin via SNAIL induction. To determine whether CDBCA-induced SNAIL induction is dependent on BMP signaling, we treated SKOV3 and OVSAHO cells in the absence and presence of CBDCA and the BMP receptor kinase inhibitors LDN193189 or RK783. The BMP inhibitors partially inhibited SNAIL induction by CBDCA (Fig. 6e), indicating that CBDCA induces SNAIL-dependent EMT partially via activation of BMP signaling in OC cells.

### CBDCA activates BMP signaling by BMP2 secretion

**Fig. 7:**
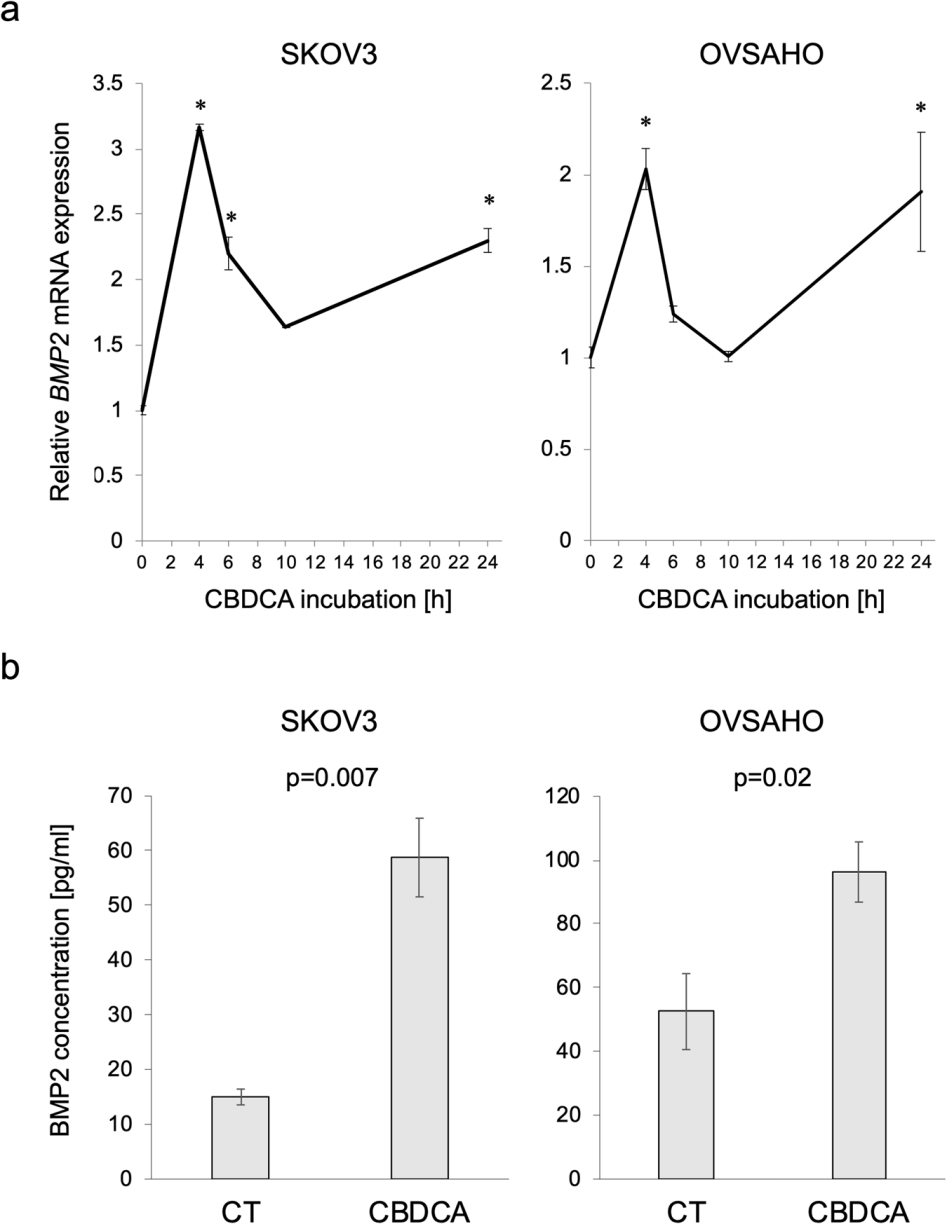
CBDCA activates BMP signaling by BMP2 secretion. **a** *BMP2* mRNA transition was monitored with RT-PCR. SKOV3 and OVSAHO cells were treated with 500 µM CBDCA for indicated periods of time. mRNA expression relative to no treatment is shown. *P-value < 0.05. **b** BMP2 secretion from OC cells were detected with ELISA. Confluent SKOV3 and OVSAHO cells were incubated in a serum-free medium with and without 500 µM CBDCA for 24 h. The conditioned medium was used for ELISA. The results are shown as the mean ± SE.

Since BMP receptor kinase inhibitors attenuated CBDCA-induced SMAD1/5/8 phosphorylation (Fig. 6e), it is possible that CBDCA activates BMP signaling upstream of BMP receptors, e.g., by inducing BMP ligands. We found that *BMP2* mRNA expression was increased after treatment with CBDCA in SKOV3 and OVSAHO cells, in a time-dependent manner (Fig. 7a). In addition, BMP2 secretion was significantly increased after CBDCA treatment of OC cells, as determined by an ELISA (Fig. 7b), suggesting that CBDCA-induced BMP2 secretion contributes to activation of BMP signaling in OC cells.

## Discussion

BMP signaling has tumor-promoting or suppressing effects depending on tumor type^12^. In this study, we demonstrate that BMP signaling has tumor-promoting effects in OC, and that BMP receptor kinase inhibitors inhibit growth, stemness, migration, and invasion of OC cells. Importantly, we have shown that CBDCA stimulates BMP2 secretion from OC cells, promoting tumorigenic autocrine stimulation.

BMP2 significantly stimulated OC cell proliferation and migration. BMP2 binds with high affinity to BMPR2 and the type I receptor ALK3, which were both expressed at high levels in the OC cells^8^. Importantly, the high expression of *BMPR2* mRNA correlated to poor prognosis for OC patients. Furthermore, BMP receptor kinase inhibitors attenuated OC cell growth at concentrations that suppressed SMAD1/5/8 phosphorylation.

BMP2 also enhanced OC cell stemness via c-KIT induction. c-KIT is a receptor tyrosine kinase, the ligand of which is stem cell factor (SCF). *SCF* mRNA expression was also induced by BMP2 (Fig. S3e). Our finding that SCF/c-KIT signaling enhanced OC stemness is consistent with previous reports^27–29^. BMP4 has been shown to modulate c-KIT expression in hematopoietic stem cells and melanocytes^30,31^. AKT is known to be activated downstream of phosphatidylinositol 3′-kinase (PI3K), which is activated by SCF binding to c-KIT^28^. Therefore, AKT phosphorylation by BMPR2 may involve activation of SCF/c-KIT signaling, in addition to the non-canonical activation of PI3K by BMP receptors.

We demonstrated that BMP2 induces EMT, migration, and invasion of OC cells, including suppression of E-cadherin expression in a SLUG-dependent manner. As E-cadherin suppression has been shown to augment OC migration and invasion, our result is consistent with previous reports^32,33^. BMP4 has been shown to induce EMT via SNAIL and SLUG in OC cells^20^. BMP2 and BMP4 may have similar functions in OC, because they both bind with high affinity to ALK3^8^. On the other hand, BMP2 did not clearly enhance the mesenchymal markers N-cadherin and vimentin. Recently, a hybrid epithelial/mesenchymal (E/M) state has been demonstrated to be most efficient in promoting cell invasion and metastasis^34^. As BMP2-stimulated OC cells expressed both epithelial and mesenchymal proteins, such a hybrid E/M state may efficiently enhance BMP2-induced OC cell migration and invasion.

Next, we demonstrated that chemotherapeutic drugs, especially CBDCA, activate BMP signaling. CBDCA-induced BMP2 secretion by OC cells. Whereas paracrine stimulation by BMP2 synthesized by OC-associated mesenchymal stem cells has been reported^35^, this is the first report which shows that autocrine BMP2 is induced after CBDCA treatment. In accordance, BMP2 expression was significantly higher in OC patients after chemotherapy than in patients without chemotherapy. The combination of CBDCA and paclitaxel is used as a first-line OC chemotherapy; CBDCA may thus induce BMP2 secretion also in OC patients. In contrast, cisplatin, another platinum-based drug, slightly suppressed SMAD1/5/8 phosphorylation of OC cells, in accordance with a previous report^36^. However, since cisplatin is known to induce a severe nephrotoxicity, the combination of CBDCA and a BMP inhibitor may be a good option for the treatment of OC.

In this study, we used two different BMP receptor kinase inhibitors, LDN193189 and RK783. Though LDN193189 is an established BMP inhibitor, its clinical use in patients is limited due to its toxicity. RK783 has recently been developed. It inhibits cell growth and phosphorylation of SMAD1/5/8 at slightly higher concentrations than LDN193189, but can be administered orally to patients and may have low toxicity and high bioavailability. A clinical trial of RK783 in fibrodysplasia ossificans progressiva (FOP) patients, with genetically enhanced BMP signaling, is planned; our findings suggest that RK783 may be used also in the treatment of OC patients.

Several reports have suggested that chemotherapy exacerbates OC cell migration and stemness^37,38^. Our results provide a possible mechanism for this observation. CBDCA activates BMP signaling, followed by SNAIL induction, and attenuation of E-cadherin expression, promoting the development of a hybrid E/M state which may lead to enhanced cell migration and stemness of OC cells. However, SNAIL induction by CBDCA was not completely reversed by BMP inhibition. Previous reports showed that also TGF-β induces SNAIL in response to cisplatin treatment of OC cells^39,40^, although CBDCA did not appreciably induce SMAD2/3 phosphorylation in OVSAHO cells. Therefore, further research will be needed to elucidate the detailed mechanism of CBDCA-induced SNAIL expression.

In order to investigate whether BMP signaling leads to chemoresistance, we established CBDCA-resistant (CBDCA-r) SKOV3 cells (Fig. S6g). Although the resistance to CBDCA was seen only at relatively low concentrations (Fig. S6h), CBDCA-r cells had higher basal SMAD1/5/8 phosphorylation (Fig. S6i) and higher sensitivity to BMP inhibitors (Fig. S6j, k). In addition, analysis of a public data set (GSE51373) revealed that chemo-resistant OC patients tended to have higher BMP2 and ID1 mRNA expression than chemo-sensitive OC patients, but it was not significant (Fig. S6l). Thus, larger trials are needed to determine whether BMP signaling contributes to chemoresistance in OC patients.

Our study has several limitations. Whereas BMP signaling has clear tumor-promoting roles in vitro, further evaluation of BMP inhibition in vivo is needed. Although RK783 inhibited OC growth in vivo, it did not clearly suppress OC dissemination in vivo. Since dissemination to organs other than the spleen rarely occurred for SKOV3 cells, other OC models are needed to definitively assess the effect of BMP signaling on OC dissemination. In addition, we showed that BMP2-induced both OC stemness and EMT. However, the exact relationship between stemness and EMT remains to be elucidated. A negative feedback loop between SCF/c-KIT and SLUG was demonstrated in hematopoietic stem cells^41^. Whether such a mechanism also occurs in OC remains to be determined. Finally, the mechanism whereby CBDCA induces BMP2 secretion from OC cells, remains to be elucidated. As *BMP2* mRNA expression was enhanced by CBDCA, increased production of BMP2 is likely to be part of the mechanism, but effects, e.g., on exocytosis, may also be involved.

In conclusion, we have demonstrated tumor-promoting effects of BMP signaling in OC, by inducing cancer stemness, EMT, migration, and invasion. We also showed that CBDCA activates BMP signaling via BMP2 secretion in OC cells. Given that CBDCA is an important drug for the treatment of OC patients, the combination of CBDCA and a BMP inhibitor could be beneficial in the treatment of OC.

## Materials and methods

### Chemicals and antibodies

CBDCA, cisplatin, paclitaxel, and imatinib were purchased from Sigma-Aldrich (St. Louis, MO, USA). LDN193189 and RK783 were synthesized at RIKEN^24^. BMP2, BMP4, BMP7, and TGF-β were purchased from R &D Systems (Minneapolis, MN, USA). Antibodies used in the present study are listed in Supplementary Table S1.

### Bioinformatic analyses

Gene expression profiles of OC samples were obtained from cBioPortal (TCGA serous ovarian cancer) and GEO database (GSE109934)^23,42^. We analyzed the TCGA serous ovarian cancer dataset, which contained RNA-Seq data of 306 patients via submitting a query to cBioPortal^23,43,44^. RNA expression cutoff Z score was adjusted to 2.0. The 306 patients were equally divided into two and three groups based on the expression of mRNA for BMP ligands and receptors, respectively. Analysis of overall survival was performed by Kaplan-Meier plots and log rank tests. A *P*-value < 0.05 was considered statistically significant. The GSE109934 dataset included 19 post-chemotherapy and 19 pre-chemotherapy samples^42^. Differentially expressed genes were identified by the GEO2R tool^45^.

### Cell culture

SKOV3, OVSAHO, OVCAR3, TOV21, OVTOKO, and A2780 OC cells were gifts from Dr. Katsutoshi Oda (The University of Tokyo, Japan). The cells were maintained in DMEM or RPMI with 10% fetal bovine serum (FBS) at 37 °C in a humidified incubator with 5% CO_2_. The cell lines were regularly tested for the absence of mycoplasma and were authenticated by identity testing.

### Gene silencing and transient transfection

Cells were cultured for 24 h before gene silencing and transfection. Stealth RNAi siRNA small interfering RNA (siRNA) for BMPR2 (HSS101065, HSS101067, HSS185979), c-KIT (HSS105820, HSS105821) and SLUG (HSS109993, HS109995) from Invitrogen, and SNAIL siRNA (sc38398) from Santa Cruz Biotechnology (Santa Cruz, CA, USA), were used. Gene silencing was performed with Lipofectamine RNAiMAX transfection reagent (Invitrogen) according to the manufacturer’s instructions. Negative controls (siNC) were from the Stealth RNAi siRNA Negative Control Kit (Invitrogen). BMPR2 expression plasmid was purchased from Addgene (Cambridge, MA, UK) and transfected into OC cells using Lipofectamine 2000 transfection reagent (Invitrogen). pcDNA 3.0 (Invitrogen) was used as a control (CT).

### Immunoblotting

Cells were harvested and soluble protein was extracted, followed by immunoblotting using the indicated antibodies, as previously described^46^. Signals were detected using a BioRad immunoblotting system (BioRad, Hercules, CA, USA) with the Immobilon Western Chemiluminescent HRP substrate (Millipore, Burlington, MA, USA). α-tubulin was used as an internal control. Approximate molecular weights of each protein were shown next to each blot.

### RNA extraction and real-time PCR

Total RNAs were extracted using the Total RNA Purification Kit (Norgen Biotek, Thoroid, Canada) according to the manufacturer’s instruction. cDNAs were synthesized from total RNAs by using the High-Capacity cDNA Reverse Transcription Kit (Applied Biosystems, San Mateo, CA, USA). Employing the CFX Connect Real-Time PCR detection system (BioRad), real-time RT-PCR fluorescence detection was performed in 96-well plates with the SYBR Green PCR Master Mix (Applied Biosystems). Primers for each gene are listed in Table S2. The threshold cycle number (Ct) for each sample was determined in triplicate. The Ct values were normalized against GAPDH.

### MTS assay

Cells were seeded into 96-well plates (3 × 10^3^ cells/well) and incubated with indicated reagents. Ten microliters of the tetrazolium salt MTS (CellTiter 96 AQueous One Solution; Promega, Madison, WI, USA) was added to each well, and the absorbance at 450 nm was monitored by using the EnSpire multimode plate reader (PerkinElmer, Waltham, MA, USA). Cell numbers were normalized relative to the absorbance of cells treated with empty plasmid, siNC, water or DMSO alone.

### Sphere formation assay

1 × 10^4^ OC cells/well were seeded into DMEM/F12 medium supplemented with 20 ng/ml EGF and 10 ng/ml bFGF from Sigma-Aldrich (St. Louis, MO, USA) in 96-well Costar ultra-low attachment plates (Corning, Corning, NY, USA) and incubated with or without 20 ng/ml BMP2 and 200 nM of the BMP receptor kinase inhibitor LDN193189 for 8 days. Total sphere numbers per well (diameters > 50 μm) were counted under microscopy.

### RNA-sequencing and gene set enrichment analysis

SKOV3 cells were cultured in 6-well plates and starved in serum-free DMEM overnight. Then cells were treated with PBS or 20 ng/ml BMP2 in DMEM containing 1% FBS for 2 h. Total RNAs were extracted as described above. Sequencing libraries for 6 samples were prepared from 1 μg total RNA using the TruSeq stranded total RNA library preparation kit with RiboZero Gold treatment (Illumina; San Diego, CA, USA) including polyA selection following the manufacturer’s instruction. Sequencing was then performed on NovaSeq SP flow cell (Illumina) by the SNP &SEQ Technology Platform at Science for Life Laboratory in Uppsala and the data has been deposited to Array Express (https://www.ebi.ac.uk/arrayexpress/) with accession number E-MTAB-9479. The data were obtained as biological triplicates. GSEA was performed with the tool available at https://www.gsea-msigdb.org/gsea/index.jsp^47^. Gene sets with a *P*-value < 0.05 and a false discovery rate (FDR) *q*-value < 0.25 were considered significant.

### Transwell invasion assay

Transwell invasion assay was performed with the 96-well Cultrex BME Cell Invasion Assay kit (R &D Systems) according to the manufacturer’s instruction. SKOV3 and OVCAR3 cells were pretreated or not with 20 ng/ml BMP2 and 200 nM LDN193189 in 1% FBS medium for 24 h. After trypsinization, 1 × 10^4^ cells in 50 μl serum-free medium were added into upper chambers precoated with Cultrex BME, and 150 μl medium containing 10% FBS were added into lower chambers. Following 24-h incubation, both chambers were washed and invaded cells were dissociated with cell dissociation/calcein-AM solution of the kit. After 1-h incubation, the absorbance at 485-nm excitation and 520-nm emission was monitored by microplate reader (PerkinElmer). The invaded cell number was calculated using a standard curve.

### Scratch assay

Cell migration was assessed by a cell culture scratch assay. SKOV3 and OVSAHO cells were cultured to confluence in 6-well plates. After scratching by 100 μl pipette tips, cells were washed with PBS and incubated with a medium containing 1% FBS in the absence or presence of 20 ng/ml BMP2 and 200 nM LDN193189 for an additional 48 h. Images were taken at 0 and 48 h after the scratch. Cell motility length was determined by measuring the width of the gap at 0 and 48 h.

### Lentiviral production

CSII-CMV-*firefly* luciferase, a lentiviral vector for the expression of *firefly* luciferase, was generated as previously described^15^. Lentiviral packaging vectors (pCAG-HIVgp and pCMV-VSV-G-Rev) were a gift from Dr. Hiroyuki Miyoshi (Keio University, Tokyo, Japan). CSII-CMV-*firefly* luciferase was transfected into HEK293FT cells together with the packaging vectors using Lipofectamine 2000 (Thermo Fisher Scientific). OC cell lines were infected with lentiviral particles.

### Animal experiments

All animal studies were approved by the Animal Experiment Committee of the Graduate School of Medicine, The University of Tokyo (Medicine-P16-140). *Firefly* luciferase gene driven by CMV promoter was lentivirally transduced into SKOV3 cells. A total of 1 × 10^4^ SKOV3 cells expressing *firefly* luciferase were injected intrabursally into the left ovary of 5-week-old female BALB/c *nu/nu* mice (Sankyo Lab Service). After 1 week of tumor engraft, mice bearing tumor were equally divided into two groups by their body weight and in vivo bioluminescence intensity. RK783 was intraperitoneally administrated at a dose of 30 mg/kg twice a day. Mice were monitored until 5 weeks post-injection. Ex vivo bioluminescence imaging was performed on NightOwl II LB983 (Berthold Technologies) by adding VivoGlo luciferin (Promega) to organs.

### Immunofluorescence

Cells were treated with indicated reagents and fixed, as previously described^46^. Cells were permeabilized in 0.2% Triton X-100 for 10 min prior to blocking in 6% bovine serum albumin (BSA) for 30 min. Cells were incubated with a primary antibody against phospho-SMAD1/5/8 (#9511, Cell Signaling Technology) at a dilution of 1:200 at 4 °C overnight, followed by incubation with a secondary antibody, Alexa Fluor 488 Goat anti-Rabbit IgG, diluted 1:200 for 1 h at room temperature. Formalin-fixed frozen sections were stained with the antibody against phospho-SMAD1/5/8 (#13820, Cell Signaling Technology) at a dilution of 1:500. Nuclei were counterstained with ProLong Gold Antifade Mountant with DAPI (Invitrogen). The cells were analyzed using a confocal fluorescence microscope (Axio Imager.M2; Carl Zeiss, Oberkochen, Germany).

### ELISA

SKOV3 and OVSAHO cells were cultured to confluence in a complete medium. After washing with PBS, cells were incubated in serum-free medium with or without 500 μM CBDCA for an additional 24 h. After the medium had been passed through a 0.45 μm syringe filter, BMP2 was quantified by a sandwich ELISA kit from Invitrogen, according to the manufacturer’s instruction.

### Statistical analysis

The experiments were repeated at least three times. Data were presented as the mean ± SE from the biological replicates for invasion assay and ELISA, whereas representative data were shown in other experiments. The significance of differences between more than three samples was analyzed by one-way ANOVA with Tukey test, whereas the significance between two samples was analyzed by two-tailed Student’s *t*-test. A *P*-value < 0.05 was considered statistically significant.

## Supplementary information

Figure S1

Figure S2

Figure S3

Figure S4

Figure S5

Figure S6

Supplementary Figure legends

Table S1

Table S2

## References

[1] Bray F (2018). Global cancer statistics 2018: GLOBOCAN estimates of incidence and mortality worldwide for 36 cancers in 185 countries. CA Cancer J. Clin..

[2] Lheureux S, Braunstein M, Oza AM (2019). Epithelial ovarian cancer: evolution of management in the era of precision medicine. CA Cancer J. Clin..

[3] Vergara D (2010). Epithelial-mesenchymal transition in ovarian cancer. Cancer Lett..

[4] Deng J (2016). Targeting epithelial-mesenchymal transition and cancer stem cells for chemoresistant ovarian cancer. Oncotarget.

[5] Markowska A, Sajdak S (2017). Role of cancer stem cells and microRNA in resistance to chemotherapy in patients with ovarian cancer. Eur. J. Gynaecol. Oncol..

[6] Loret, N., Denys, H., Tummers, P. & Berx, G. The role of epithelial-to-mesenchymal plasticity in ovarian cancer progression and therapy resistance. *Cancers* **11**, 10.3390/cancers11060838 (2019). 10.3390/cancers11060838 PMC6628067 31213009

[7] Mihanfar A (2019). Ovarian cancer stem cell: a potential therapeutic target for overcoming multidrug resistance. J. Cell Physiol..

[8] Miyazono K, Kamiya Y, Morikawa M (2010). Bone morphogenetic protein receptors and signal transduction. J. Biochem..

[9] Shepherd TG, Nachtigal MW (2003). Identification of a putative autocrine bone morphogenetic protein-signaling pathway in human ovarian surface epithelium and ovarian cancer cells. Endocrinology.

[10] Shepherd TG, Thériault BL, Nachtigal MW (2008). Autocrine BMP4 signalling regulates ID3 proto-oncogene expression in human ovarian cancer cells. Gene.

[11] Ho CC, Zhou X, Mishina Y, Bernard DJ (2011). Mechanisms of bone morphogenetic protein 2 (BMP2) stimulated inhibitor of DNA binding 3 (Id3) transcription. Mol. Cell Endocrinol..

[12] Davis H, Raja E, Miyazono K, Tsubakihara Y, Moustakas A (2016). Mechanisms of action of bone morphogenetic proteins in cancer. Cytokine Growth Factor Rev..

[13] Piccirillo SG (2006). Bone morphogenetic proteins inhibit the tumorigenic potential of human brain tumour-initiating cells. Nature.

[14] Lee J (2008). Epigenetic-mediated dysfunction of the bone morphogenetic protein pathway inhibits differentiation of glioblastoma-initiating cells. Cancer Cell.

[15] Raja E (2017). Bone morphogenetic protein signaling mediated by ALK-2 and DLX2 regulates apoptosis in glioma-initiating cells. Oncogene.

[16] Deng H, Ravikumar TS, Yang WL (2007). Bone morphogenetic protein-4 inhibits heat-induced apoptosis by modulating MAPK pathways in human colon cancer HCT116 cells. Cancer Lett..

[17] Yokoyama Y (2017). Autocrine BMP-4 signaling is a therapeutic target in colorectal cancer. Cancer Res..

[18] Le Page C (2009). BMP-2 signaling in ovarian cancer and its association with poor prognosis. J. Ovarian Res..

[19] Peng J (2016). The BMP signaling pathway leads to enhanced proliferation in serous ovarian cancer-A potential therapeutic target. Mol. Carcinog..

[20] Thériault BL, Shepherd TG, Mujoomdar ML, Nachtigal MW (2007). BMP4 induces EMT and Rho GTPase activation in human ovarian cancer cells. Carcinogenesis.

[21] Coffman LG (2016). Human carcinoma-associated mesenchymal stem cells promote ovarian cancer chemotherapy resistance via a BMP4/HH signaling loop. Oncotarget.

[22] Herrera B, van Dinther M, ten Dijke P, Inman GJ (2009). Autocrine bone morphogenetic protein-9 signals through activin receptor-like kinase-2/Smad1/Smad4 to promote ovarian cancer cell proliferation. Cancer Res..

[23] Integrated genomic analyses of ovarian carcinoma. *Nature* **474**, 609–615 (2011). 10.1038/nature10166 PMC3163504 21720365

[24] Yu PB (2008). BMP type I receptor inhibition reduces heterotopic [corrected] ossification. Nat. Med..

[25] Zhang S (2008). Identification and characterization of ovarian cancer-initiating cells from primary human tumors. Cancer Res..

[26] Steg AD (2012). Stem cell pathways contribute to clinical chemoresistance in ovarian cancer. Clin. Cancer Res..

[27] Chau WK, Ip CK, Mak AS, Lai HC, Wong AS (2013). c-Kit mediates chemoresistance and tumor-initiating capacity of ovarian cancer cells through activation of Wnt/β-catenin-ATP-binding cassette G2 signaling. Oncogene.

[28] Figueira MI, Cardoso HJ, Correia S, Maia CJ, Socorro S (2017). The stem cell factor (SCF)/c-KIT system in carcinogenesis of reproductive tissues: what does the hormonal regulation tell us?. Cancer Lett..

[29] Mazzoldi EL (2019). A juxtacrine/paracrine loop between C-Kit and stem cell factor promotes cancer stem cell survival in epithelial ovarian cancer. Cell Death Dis..

[30] Marshall CJ, Sinclair JC, Thrasher AJ, Kinnon C (2007). Bone morphogenetic protein 4 modulates c-Kit expression and differentiation potential in murine embryonic aorta-gonad-mesonephros haematopoiesis in vitro. Br. J. Haematol..

[31] Kawakami T (2008). BMP-4 upregulates Kit expression in mouse melanoblasts prior to the Kit-dependent cycle of melanogenesis. J. Invest. Dermatol..

[32] Sawada K (2008). Loss of E-cadherin promotes ovarian cancer metastasis via alpha 5-integrin, which is a therapeutic target. Cancer Res..

[33] Rosso M (2017). E-cadherin: a determinant molecule associated with ovarian cancer progression, dissemination and aggressiveness. PLoS ONE.

[34] Tsubakihara, Y. & Moustakas, A. Epithelial-mesenchymal transition and metastasis under the control of transforming growth factor β. *Int. J. Mol. Sci.*10.3390/ijms19113672 (2018). 10.3390/ijms19113672 PMC6274739 30463358

[35] McLean K (2011). Human ovarian carcinoma–associated mesenchymal stem cells regulate cancer stem cells and tumorigenesis via altered BMP production. J. Clin. Invest..

[36] Hover LD (2015). Small molecule inhibitor of the bone morphogenetic protein pathway DMH1 reduces ovarian cancer cell growth. Cancer Lett..

[37] Rizzo S (2011). Ovarian cancer stem cell-like side populations are enriched following chemotherapy and overexpress EZH2. Mol. Cancer Ther..

[38] Zhao Y (2020). Chemotherapy exacerbates ovarian cancer cell migration and cancer stem cell-like characteristics through GLI1. Br. J. Cancer.

[39] Zhu H (2018). A novel TGFβ trap blocks chemotherapeutics-induced TGFβ1 signaling and enhances their anticancer activity in gynecologic cancers. Clin. Cancer Res..

[40] Sonego M (2019). USP1 links platinum resistance to cancer cell dissemination by regulating Snail stability. Sci. Adv..

[41] Zhang Z (2017). A novel slug-containing negative-feedback loop regulates SCF/c-Kit-mediated hematopoietic stem cell self-renewal. Leukemia.

[42] Arend RC (2018). Molecular response to neoadjuvant chemotherapy in high-grade serous ovarian carcinoma. Mol. Cancer Res..

[43] Cerami E (2012). The cBio cancer genomics portal: an open platform for exploring multidimensional cancer genomics data. Cancer Discov..

[44] Gao J (2013). Integrative analysis of complex cancer genomics and clinical profiles using the cBioPortal. Sci. Signal.

[45] Davis S, Meltzer PS (2007). GEOquery: a bridge between the Gene Expression Omnibus (GEO) and BioConductor. Bioinformatics.

[46] Fukuda T (2015). The anti-malarial chloroquine suppresses proliferation and overcomes cisplatin resistance of endometrial cancer cells via autophagy inhibition. Gynecol. Oncol..

[47] Mootha VK (2003). PGC-1alpha-responsive genes involved in oxidative phosphorylation are coordinately downregulated in human diabetes. Nat. Genet..

