# Supplementary figures and images for: BMP signaling is a therapeutic target in ovarian cancer
Source: Cell Death Discov. 2020 Dec 5;6:139. doi: 10.1038/s41420-020-00377-w (PMC7719168; doi:10.1038/s41420-020-00377-w)

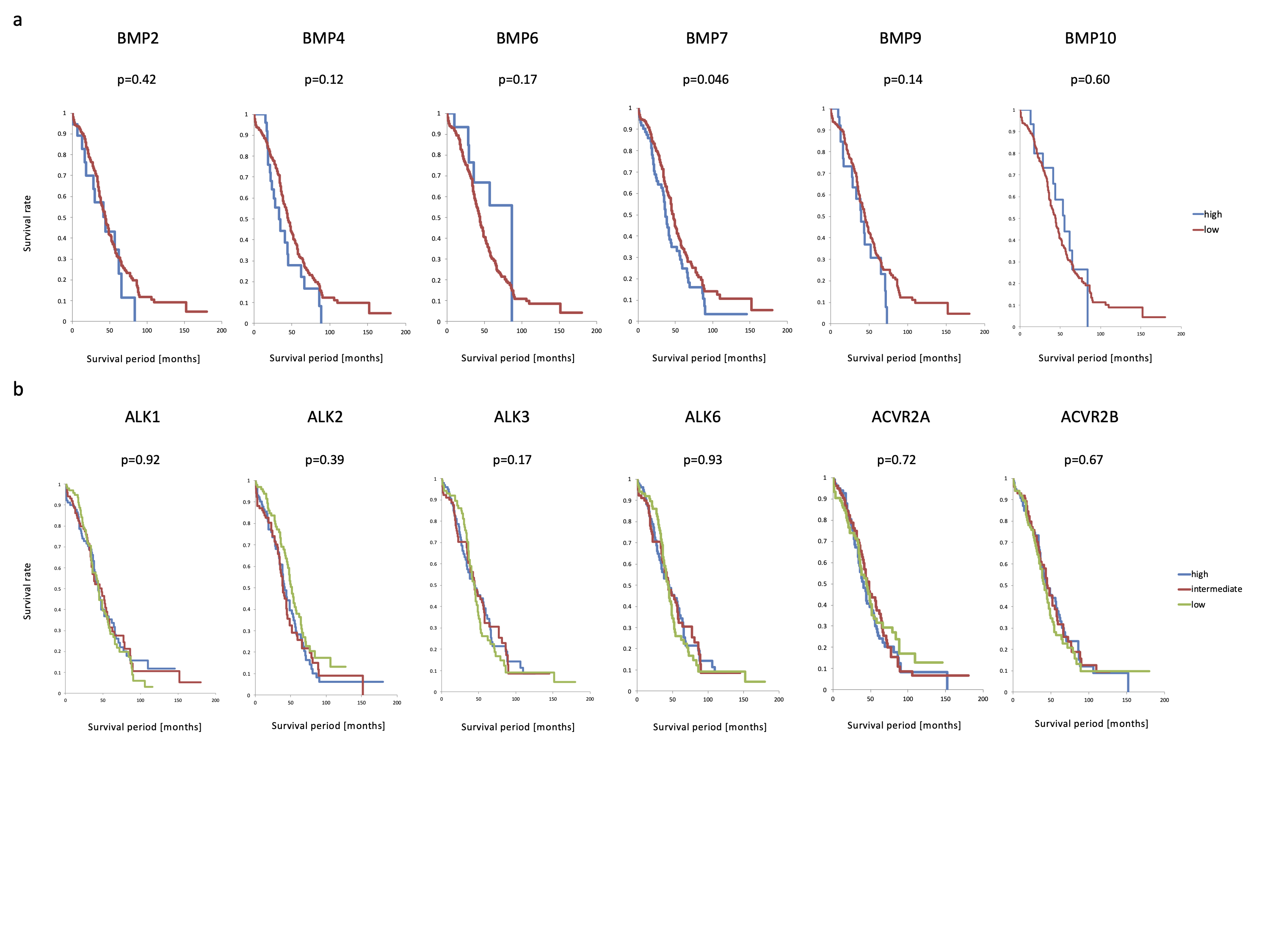

Supplement: Supplementary file 1 — Figure S1 [file 41420_2020_377_MOESM1_ESM.png]

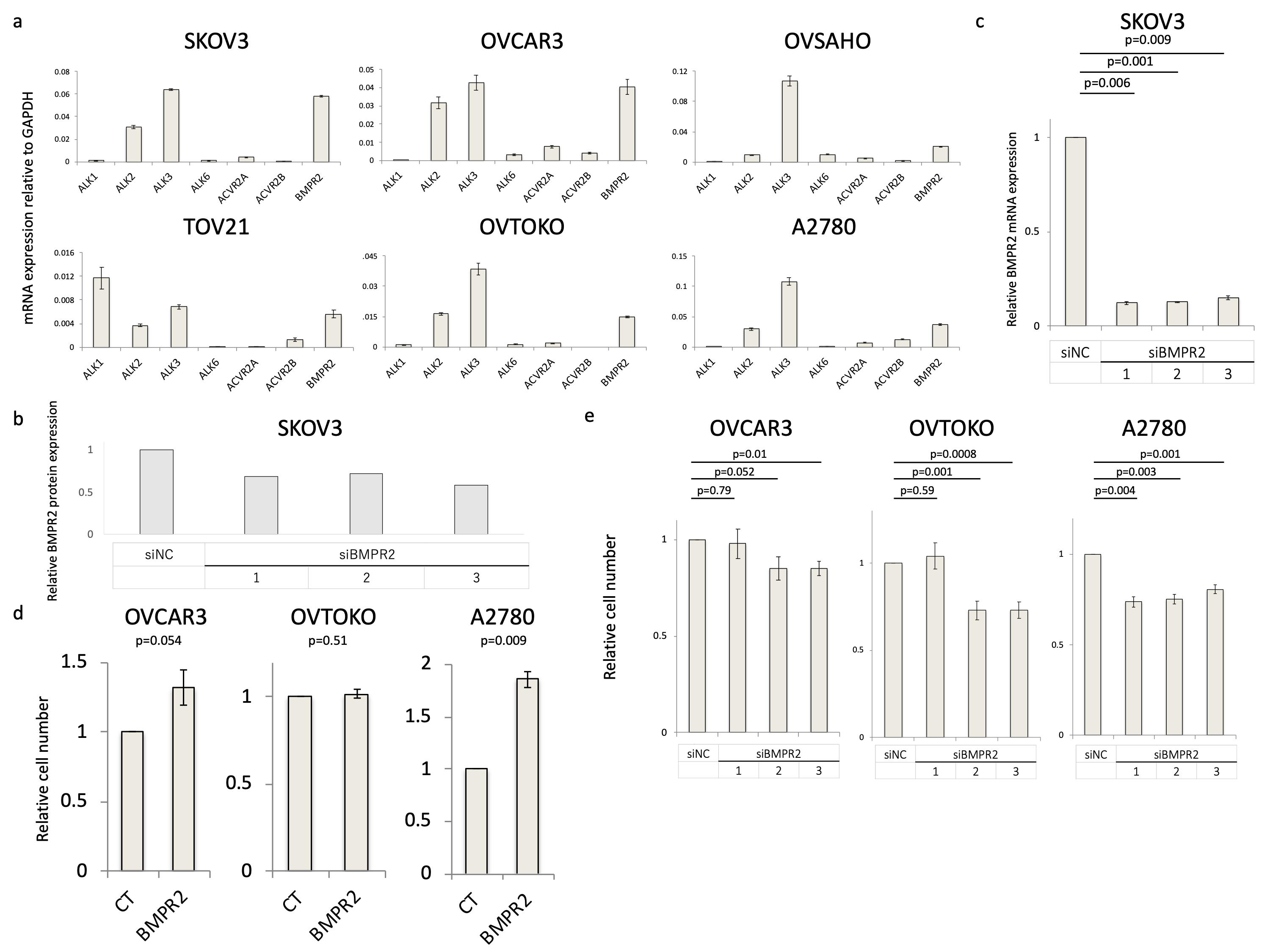

Supplement: Supplementary file 2 — Figure S2 [file 41420_2020_377_MOESM2_ESM.png]

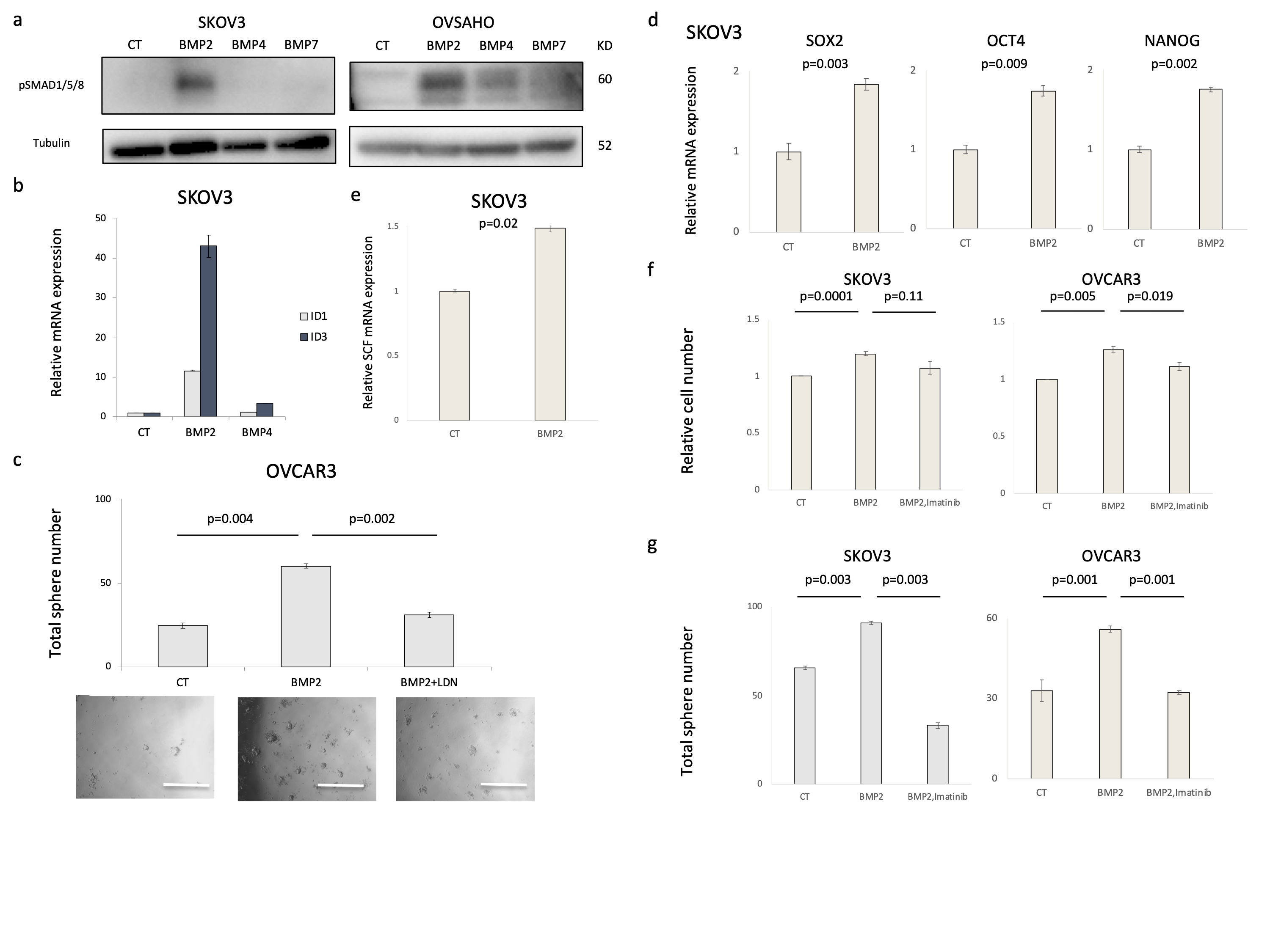

Supplement: Supplementary file 3 — Figure S3 [file 41420_2020_377_MOESM3_ESM.png]

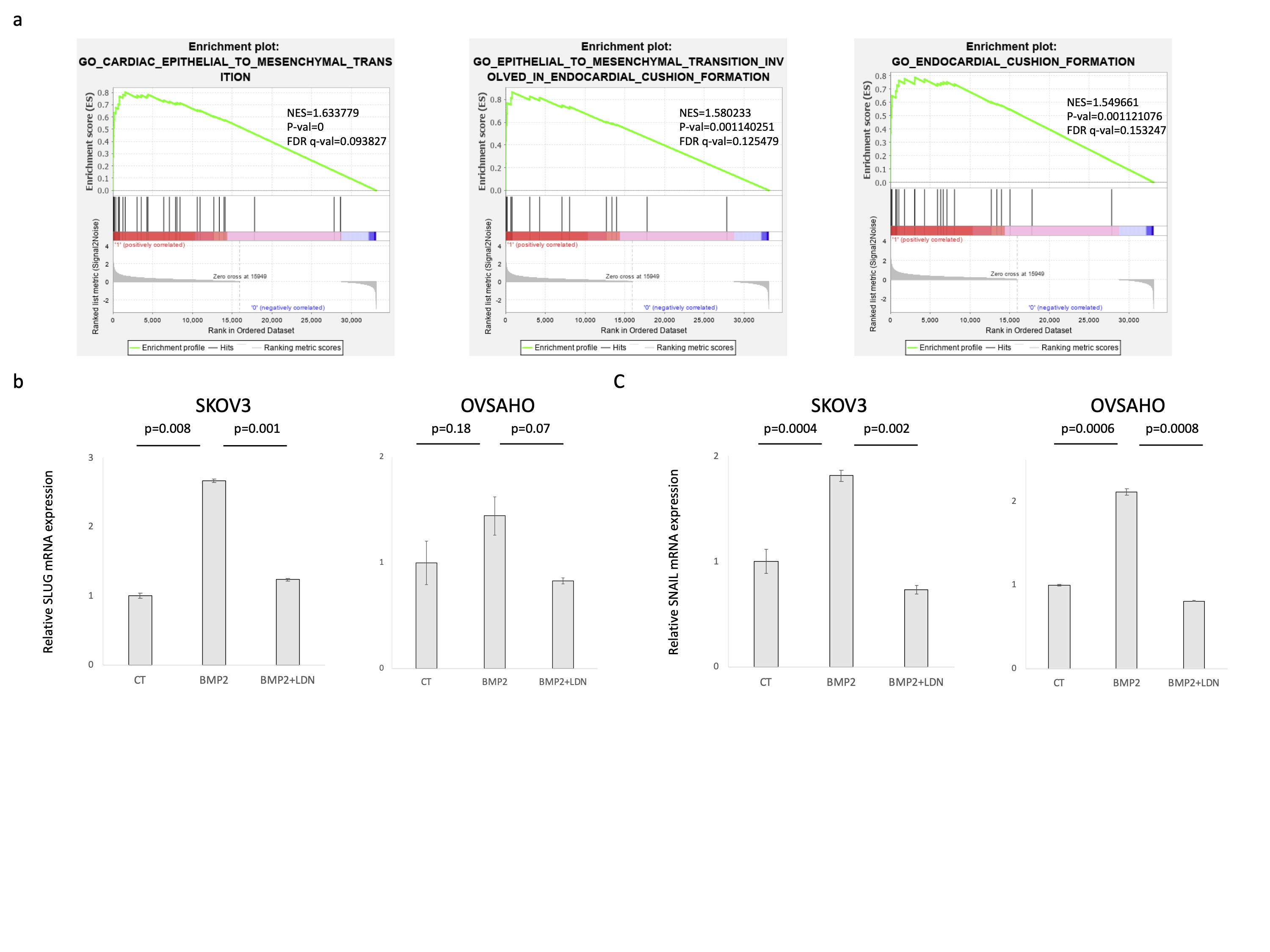

Supplement: Supplementary file 4 — Figure S4 [file 41420_2020_377_MOESM4_ESM.png]

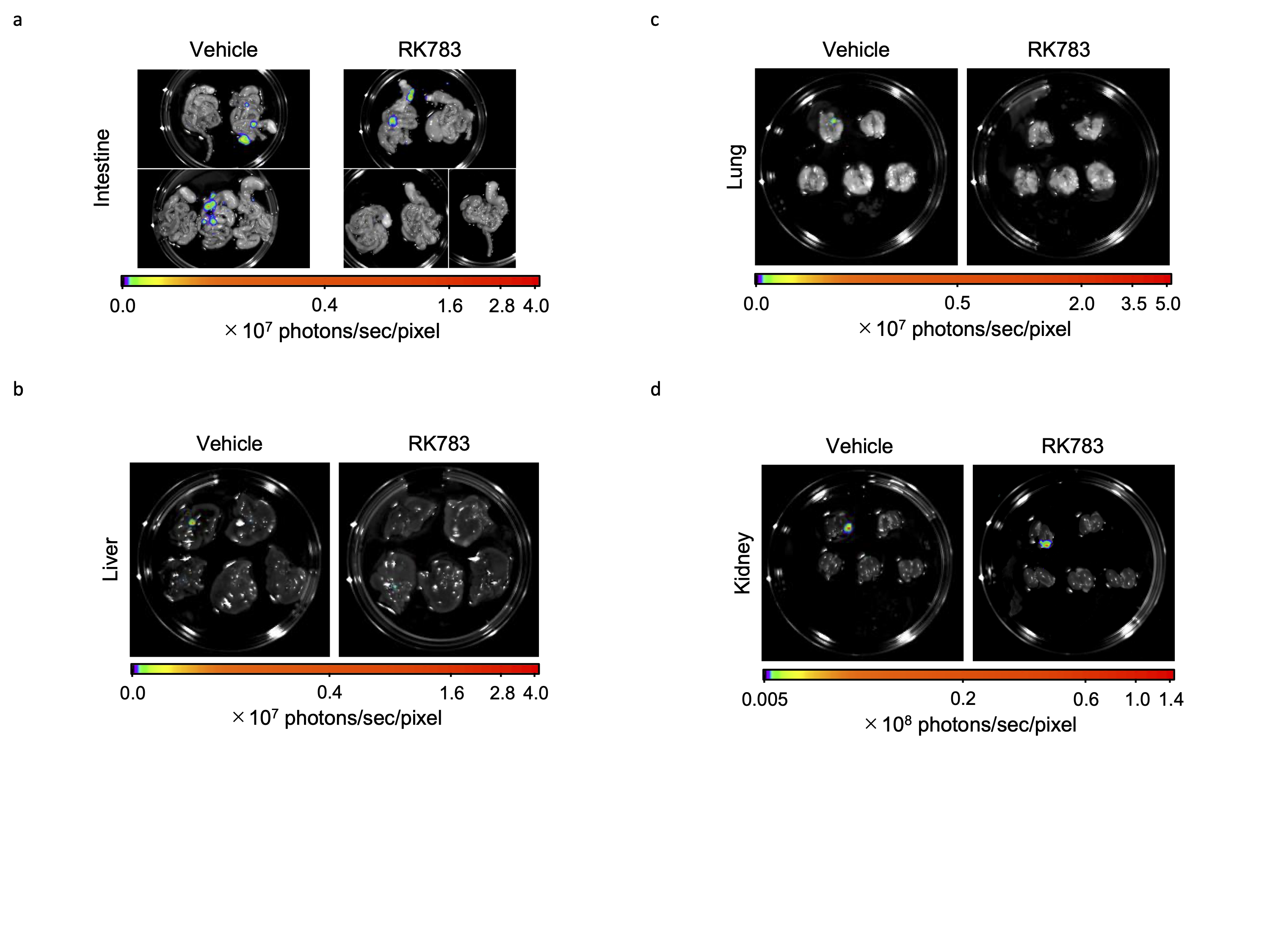

Supplement: Supplementary file 5 — Figure S5 [file 41420_2020_377_MOESM5_ESM.png]

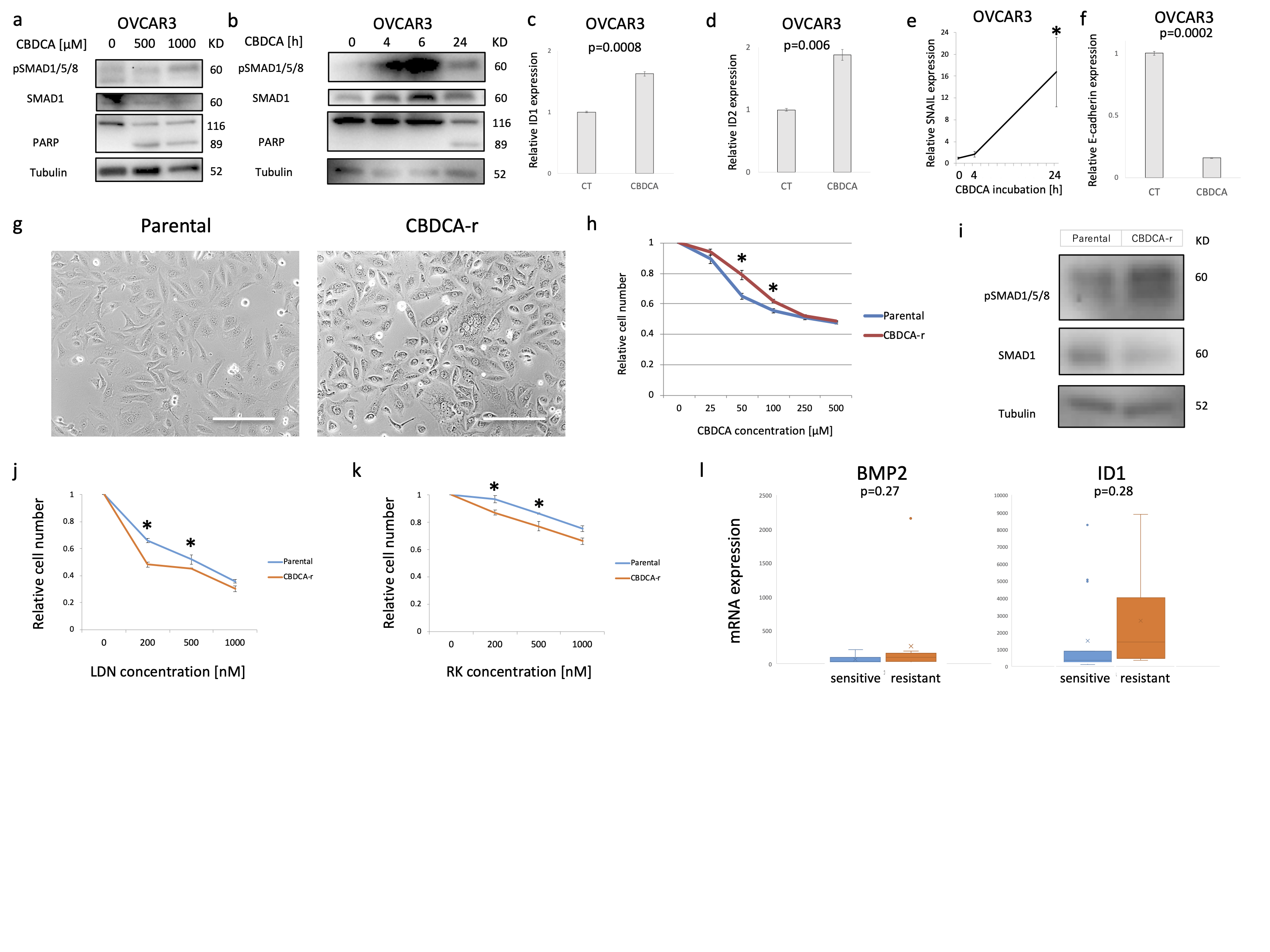

Supplement: Supplementary file 6 — Figure S6 [file 41420_2020_377_MOESM6_ESM.png]
