# Supplementary material for: BMP signaling is a therapeutic target in ovarian cancer
Source: Cell Death Discov. 2020 Dec 5;6:139. doi: 10.1038/s41420-020-00377-w (PMC7719168; doi:10.1038/s41420-020-00377-w)
Supplement: Supplementary file 7 — Supplementary Figure legends [file 41420_2020_377_MOESM7_ESM.docx]

**Supplementary Figure Legends**

**Figure S1.** **Correlation between BMP ligands or receptors and overall survival of 306 OC patients.**

(a) Correlation between mRNA expression of BMP ligands and overall survival of 306 OC patients’ data derived from TCGA serous ovarian cancer.

(b) Correlation between mRNA expression of BMP receptors and overall survival of 306 OC patients’ data derived from TCGA serous ovarian cancer.

Based on mRNA expression, the 306 patients were equally divided into two (high, low) groups for panel (a) or three (high, intermediate, low) groups for panel (b). A *P*-value was calculated with a log-rank test.

**Figure S2.** **Expression of BMP receptors in OC cell lines, and the effects of BMPR2 on cell proliferation.**

(a) mRNA expression levels of BMP receptors were evaluated by real-time PCR in six OC cell lines. mRNA expression was normalized relative to GAPDH. (b,c) *BMPR2* protein (b) and mRNA (c) suppression by three different siBMPR2 oligonucleotides was assessed by IB and real-time PCR, respectively, in SKOV3 cells. Protein expression was normalized relative to α-tubulin. (d) MTS assay was performed to monitor cell proliferation after plasmid transfection in three OC cells. (e) Cell proliferation was evaluated by MTS assay with siRNA transfection in three OC cells. Cells were treated as previously mentioned. The results in panels (c)-(e) are shown as the mean ± SE.

**Figure S3.** **BMP2 enhances OC cell proliferation, sphere formation and stemness.**

(a) SKOV3 and OVSAHO cells were treated with 20 ng/ml BMP2, 20 ng/ml BMP4 or 30 ng/ml BMP7 for 24 h. IB was used to monitor SMAD1/5/8 phosphorylation. (b) *ID1/3* mRNA was assessed by real-time PCR after 2 h stimulation of 20 ng/ml BMP2 or 20 ng/ml BMP4 in SKOV3 cells. Relative mRNA expression to CT (PBS). (c) Sphere formation assay of OVCAR3 cells. (d,e) Indicated mRNA expression was determined after 20 ng/ml BMP2 treatment for 48 h in SKOV3 cells. mRNA expression is relative to CT (PBS). (f,g) Cell proliferation and stemness were assessed by MTS and sphere formation assay in SKOV3 and OVCAR3 cells. Cells were treated with PBS or 20 ng/ml BMP2 or BMP2+5 μM Imatinib for 3 and 8 days respectively. The results in panels (b)-(g) are shown as the mean ± SE.

**Figure S4.** **EMT-like phenotypes of SKOV3 cells and induction of *SLUG* and *SNAIL* by BMP2.**

(a) GSEA of RNA-seq was performed in SKOV3 cells. Cells treated with 20 ng/ml BMP2 for 2 h were compared with PBS-treated cells. Indicated enrichment plots are shown. (b, c) *SLUG* (b) and *SNAIL* (c) mRNA were monitored by real-time PCR in SKOV3 and OVSAHO cells. These cells were incubated with PBS (CT), 20 ng/ml BMP2 or BMP2+LDN for 72 h. mRNA expression was normalized relative to CT. The results in panels (b) and (c) are shown as the mean ± SE.

**Figure S5.** **Effects of RK783 on OC cell metastases *in vivo*.**

Ex vivo bioluminescent imaging of intestine (a), lung (b), liver (c) and kidney (d) derived from the orthotopic xenograft (n = 5 mice per group) of SKOV3 cells.

**Figure S6.** **Chemotherapy activates BMP signaling in OC cells as well as in chemotherapy-resistant OC cells.**

(a) OVCAR3 cells were treated with CBDCA at indicated concentrations for 24 h. IB was used to detect protein expression. (b) OVCAR3 cells were incubated with 1 mM CBDCA for indicated time periods. Protein expression was assessed by IB. (c, d, e, f) Real-time PCR was used to monitor *ID1*, *ID2*, *SNAIL* and *E-cadherin* mRNA expression in OVCAR3 cells. OVCAR3 cells were treated with 1 mM CBDCA for 4 h (ID1/2, SNAIL) and 24 h (SNAIL, E-cadherin). Expression relative to no treatment is shown. (g, h) Establishment of CBDCA-resistant SKOV3 cells (CBDCA-r). Micrographs of the cell morphology are shown (g). Scale bar = 100 μm. Parental SKOV3 cells were incubated with complete medium containing CBDCA for three months. CBDCA concentration was gradually increased up to 30 μM with each passage. After 1 month of incubation with 30 μM CBDCA, cells were cultured in medium without CBDCA for 1 month and the sensitivity to CBDCA was determined by MTS assay (h). (i) Protein expression of phospho-SMAD1/5/8 and SMAD1 was compared between parental and CBDCA-r SKOV3 cells by IB. (j, k) MTS assay was performed to determine the sensitivity to LDN193189 (LDN) (j) and RK783 (RK) (k) in parental and CBDCA-r SKOV3 cells. Cells were incubated with LDN or RK for 72 h. (l) GSE51373 dataset from GEO database was analyzed. The dataset included microarray results of 16 chemo-sensitive (sensitive) and 12 chemo-resistant (resistant) OC patients. BMP2 and ID1 expression was compared between the two groups, using the GEO2R tools. The results in panels (c)-(f) are shown as the mean ± SE. **P*-value < 0.05
